# Older Age and Time to Medical Assistance Are Associated with Severity and Mortality of Snakebites in the Brazilian Amazon: A Case-Control Study

**DOI:** 10.1371/journal.pone.0132237

**Published:** 2015-07-13

**Authors:** Esaú L. Feitosa, Vanderson S. Sampaio, Jorge L. Salinas, Amanda M. Queiroz, Iran Mendonça da Silva, André A. Gomes, Jacqueline Sachett, André M. Siqueira, Luiz Carlos L. Ferreira, Maria Cristina dos Santos, Marcus Lacerda, Wuelton Monteiro

**Affiliations:** 1 Departamento de Ensino e Pesquisa, Fundação de Medicina Tropical Doutor Heitor Vieira Dourado, Manaus, Brazil; 2 Escola Superior de Ciências da Saúde, Universidade do Estado do Amazonas, Manaus, Brazil; 3 Núcleo de Sistemas de Informação, Fundação de Vigilância em Saúde do Amazonas, Manaus, Brazil; 4 Department of Medicine, Emory University, Atlanta, United States of America; 5 Instituto Nacional de Infectologia Evandro Chagas, Fundação Oswaldo Cruz, Rio de Janeiro, Brazil; 6 Instituto de Ciências Biológicas, Universidade Federal do Amazonas, Manaus, Brazil; 7 Instituto Leônidas & Maria Deane, Fundação Oswaldo Cruz, Manaus, Brazil; Universidad de Costa Rica, COSTA RICA

## Abstract

The Amazon region reports the highest incidence of snakebite envenomings in Brazil. We aimed to describe the epidemiology of snakebites in the state of Amazonas and to investigate factors associated with disease severity and lethality. We used a nested case-control study, in order to identify factors associated with snakebite severity and mortality using official Brazilian reporting systems, from 2007 to 2012. Patients evolving to severity or death were considered cases and those with non-severe bites were included in the control group. During the study period, 9,191 snakebites were recorded, resulting in an incidence rate of 52.8 cases per 100,000 person/years. Snakebites mostly occurred in males (79.0%) and in rural areas (70.2%). The most affected age group was between 16 and 45 years old (54.6%). Fifty five percent of the snakebites were related to work activities. Age ≤15 years [OR=1.26 (95% CI=1.03-1.52); (p=0.018)], age ≥65 years [OR=1.53 (95% CI=1.09-2.13); (p=0.012)], work related bites [OR=1.39 (95% CI=1.17-1.63); (p<0.001)] and time to medical assistance >6 hours [OR=1.73 (95% CI=1.45-2.07); (p<0.001)] were independently associated with the risk of severity. Age ≥65 years [OR=3.19 (95% CI=1.40-7.25); (p=0.006)] and time to medical assistance >6 hours [OR=2.01 (95% CI=1.15-3.50); (p=0.013)] were independently associated with the risk of death. Snakebites represent an occupational health problem for rural populations in the Brazilian Amazon with a wide distribution. These results highlight the need for public health strategies aiming to reduce occupational injuries. Most cases of severe disease occurred in the extremes of age, in those with delays in medical attention and those caused by *Micrurus* bites. These features of victims of snakebite demand adequate management according to well-defined protocols, including prompt referral to tertiary centres when necessary, as well as an effective response from surveillance systems and policy makers for these vulnerable groups.

## Introduction

Snakebites represent a treatable health problem affecting predominantly tropical low- and middle-income countries [1,2]. The annual number of snakebite cases worldwide is largely unknown and probably underreported with estimates that could reach 5.5 million snakebites annually, with 94,000 estimated deaths [1]. Rural areas in developing countries are the most affected, with varying seasonality usually characterized by peak incidences seen in the rainy and harvesting seasons [3]. The burden of human suffering caused by snakebite has not received the attention it deserves from the public health community, development agencies and governments, and may therefore be appropriately categorized as a neglected tropical disease [1,4]. Most snakebite reporting systems are fragile and underestimate the actual injury burden [5]. Better epidemiological surveillance is necessary to assess the extent of this important public health problem to improve prevention and treatment measures. Most deaths and sequelae from snakebites are preventable by interventions such as making antivenom widely available [6].

In 2013, the Brazilian Ministry of Health reported 27,181 snakebite cases [7]. The highest incidence was observed in the Brazilian Amazon (52.6 cases/100,000 inhabitants) followed by the Midwest (16.4/100,000). These values are expected to be higher in remote areas of the Brazilian Amazon [8] due to considerable underreporting. In this region, adult males living in rural areas [3,9–13] and/or workers exerting farming, hunting and forestry activities [14,15] are the most affected groups, strongly suggesting an occupational risk. A community survey conducted with forest-dwelling Indians and rubber tappers (*seringueiros*) revealed that 13% of the surveyed population had been bitten during their lifetime [14]. Snakebite incidence correlates with the period of higher rainfalls as increased river levels force snakes to seek new shelters closer to human settlements [3,9,11,12], highlighting the vulnerability of Amazonian communities living in riverbank areas.

Some studies indicate that snakes of the genus *Bothrops* account for nearly 80% of the snakebites in the Brazilian Amazon region [16–18]. *Bothrops atrox* and *Lachesis muta* share the same popular name ‘*surucucu*’ in certain Amazonian areas, bringing a confounding factor in snake identification by the local population [12]. In the Amazon, clinical data from *Bothrops atrox* bites show pain as the most frequent local manifestation, followed by swelling, warmth on palpation and necrosis [18,19]. Around 25% of the patients present systemic manifestations, including spontaneous systemic bleeding, ranging from 16–18% [16,18,19] and acute renal failure registered in 10.9% in a case series [19]. A total of 39.0% of patients developed secondary complications, such as cellulitis and abscesses [18]. The clinical picture caused by *Lachesis* bites is very similar to *Bothrops’*, except for vagal symptoms that may be present especially in severe *Lachesis* cases [7,8]. In the Brazilian Amazon, relatively few accidents are caused by coral snakes (*Micrurus* sp.) [20] or rattlesnakes (*Crotalus* sp.) [21]. Surveillance systems shows an overall case-fatality rate ranging from 0.4 to 3.9% in the Brazilian Amazon [9,11,12,14,15].

Snakebite clinical severity varies by snake species and patient characteristics, but there are few studies identifying the more vulnerable subgroups developing poor outcomes. In India, patient-related factors such as child age, previous health condition and time elapsed until medical assistance can act as risk factors for severity, sequelae and death [22–26]. In the same country, patients admitted with symptoms of neurotoxicity and vomiting [22,24], coagulopathy and leukocytosis [23], hypertension, albuminuria, changes in bleeding and prothrombin time [25] were more likely to develop severe outcomes. The delay in antivenom administration was also a risk factor for severity in Nigeria [27]. Capillary leak syndrome, bleeding and respiratory paralysis were risk factors for mortality in patients who received snake antivenom in Nigeria and Korea [28,29].

A better knowledge of severity and mortality due to snakebite would lead to improved management, with expected reduction of sequelae and lethality rates in remote localities in the Brazilian Amazon. The aim of this study is to analyze the profile of snakebites reported in the state of Amazonas, in the Western Brazilian Amazon, and to investigate potential risk factors for severity and lethality.

## Materials and Methods

### Study area

The State of Amazonas is located in the western Brazilian Amazon, comprising an area of 1,570,946.8 km^2^, with 62 municipalities. The estimated population of the state was 3,807,921 inhabitants in 2010, with 74.2% living in urban zones and 25.8% in rural areas. Approximately 45% of the population lives in the state capital, Manaus. The state has a reduced coverage of highways and roads, with much of the displacement happening via river transportation. The state is densely covered by an evergreen rain forest, standing out the upland forests (*terra firme* forest), floodplains (*várzeas*) and flooded areas (*igapós*).

### Data source

Snakebites are compulsorily recorded by the Brazilian Notifiable Diseases Surveillance System [*Sistema de Informação de Agravos de Notificação* (SINAN)] based on data report forms used in the investigation and follow-up of cases of venomous animals. The SINAN is a national electronic surveillance system that contains a variety of diseases in an integrated database that also includes snakebites [15]. We included all snakebite cases in the state of Amazonas reported to SINAN from January 1, 2007 to December 31, 2012. Snakebite treatment is provided free of charge only by the Brazilian Government and is not available for purchase in the private sector.

### Exposure and outcome definitions

Reporting on snakebites’ severity grading and outcomes (discharge or death) is required by SINAN and it is entered by healthcare providers at the time of case notification (which often happens after discharge). Clinical severity of snakebites in this work was classified according the Brazilian Health Ministry guidelines [17]: i) **mild cases**: local pain, local swelling and bruising for *Bothrops* and *Lachesis* bites or ptosis, slight blurred vision and mild or absent myalgia for *Crotalus* bites (rarely found in the study area) [21]; ii) **moderate cases**: local manifestations without necrosis, minor systemic signs (coagulopathy and bleeding, no shock) in *Bothrops* and *Lachesis* bites; myalgia and dark urine in may occur in *Crotalus* bites; iii) **severe cases**: life-threatening snakebite, with severe bleeding, hypotension/shock and/or acute renal failure for *Bothrops* and *Lachesis* bites. In severe *Lachesis* bites, vagal symptoms may be present in about 14% of cases [30]. Severe *Crotalus* are those presenting with generalized myalgia, dark urine, oliguria or anuria. It is recommended to consider all confirmed *Micrurus* cases, although scarcely seen in the Amazonas, as potentially severe due to the risk of respiratory failure. For more details concerning the severity grading and treatment of snakebites used in Brazil see S1 File.

The variables analyzed were sex, age (in years), anatomical region of the bite, area of occurrence (rural or urban), work-related injury (yes or no), time elapsed between the bite and medical assistance (in hours), perpetrating species (*Bothrops*, *Lachesi*s, *Crotalus*, *Micrurus* or non-venomous snakes), severity grading (mild, moderate or severe), outcome (discharge or death), clinical features and antivenom administration data. All variables were checked by two independent researchers before analysis and further investigated for a possible association with severity and lethality as dependent variables.

Due to the potential severity of *Lachesis* confirmed bites, these few cases are generally considered clinically moderate or severe. However, in areas where there is overlap in the geographical distribution of *Bothrops* and *Lachesis*, such as in the Brazilian Amazon, the differential routine diagnosis when the snake has not been captured can be misleading and be associated with misclassification of the causing species.

In order to identify factors associated with snakebite severity and mortality, a nested case control study was used wherein patients evolving to severity or death were classified as cases and those with mild and moderate bites were included as controls.

### Spatial distribution

A map was created with the software ArcMap 10.1 in ArcGIS 10.1 (ESRI, USA) using estimates of the mean incidence by municipality. The incidence rate was calculated dividing cases by the population of each municipality [31] by 100 thousand inhabitants. Spatial interpolation of snakebites’ incidence was mapped using data from 62 municipalities by the Inverse Distance Weighting (IDW) method.

### Statistical analysis

Only variables presenting completeness higher than 85% were analyzed. Duplicity was solved before data analysis. Data were analyzed using SPSS version 21.0 for Windows (SPSS Inc. Chicago, IL, USA). Proportions of severe cases and deaths were compared by Chi-square test (corrected by Fisher' test if necessary); differences were considered statistically significant for p<0.05. The crude *Odds Ratio* (OR) with its respective 95% Confidence Interval (95% CI) was determined considering severity and death as the dependent variables. Logistic regression was used for the multivariable analyses and the adjusted *Odds Ratios* with 95% CI were also calculated. All variables associated with the outcomes at a significance level of p<0.20 in the univariable analysis were included in the multivariable analysis. Statistical significance was considered if p<0.05 in the Hosmer-Lemeshow goodness-of-fit test.

### Ethical clearance

This study was approved by the Ethics Review Board (ERB) of the *Fundação de Medicina Tropical Dr*. *Heitor Vieira Dourado* (approval number 872.520/2014), as well as by the ERB of the Health Surveillance Foundation of the Amazonas State. All data analyzed were anonymous. Since data were obtained exclusively from surveillance databases, the ERB gave a waiver of informed consent.

## Results

### Study population

**Fig 1:**
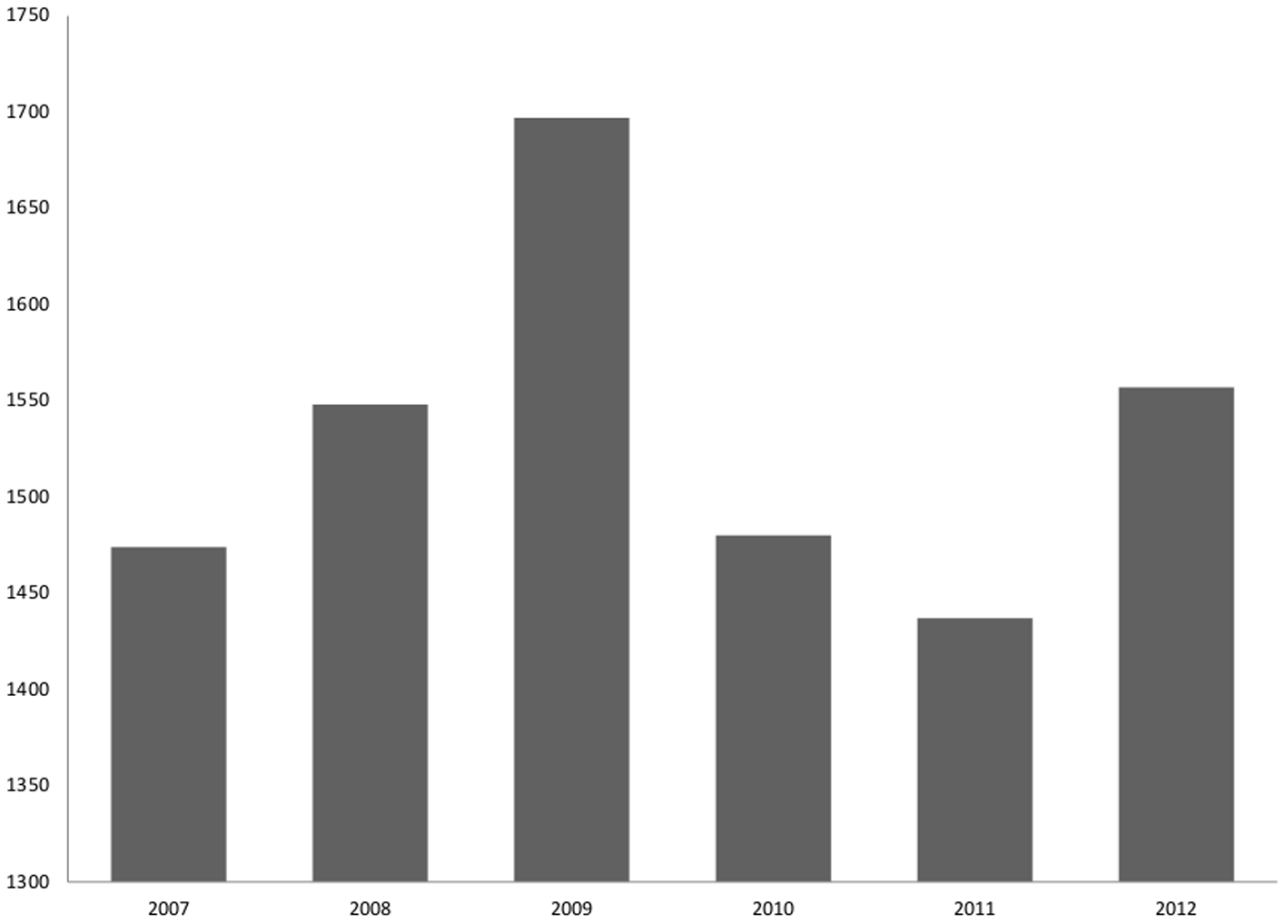
Absolute number of snakebite envenomings in the state of Amazonas, reported by year, from 2007 to 2012. Incidence was slightly higher in 2009 (1,697 cases; 58.5 per 100,000 inhabitants) and 2012 (1,558 cases; 53.7 per 100,000) and lower in 2011 (1,436 cases; 49.5 per 100,000).

According to the official reporting systems, 9,191 snakebites were recorded in the Amazonas State from 2007 to 2012, resulting in an incidence rate of 52.8 cases per 100,000 person/year. There was a slight variation in the annual incidence rates during the study period (Fig 1). Incidence was higher in 2009 (1,697 cases; 58.5 per 100,000 inhabitants) and 2012 (1,558 cases; 53.7 per 100,000) and lower in 2011 (1,436 cases; 49.5 per 100,000). All the variables retrieved from the original dataset presented completeness higher than 85% (Table 1). Most of the snakebites occurred in males (7,257 cases; 79.0%). Regarding the area of occurrence, 70.2% were reported in rural areas. The most affected age group was between 16 and 45 years old (5,019 cases; 54.6%). A proportion of 55.0% of the snakebites were related to work activities. Regarding time elapsed from the bite until medical assistance, 68.4% of the cases received treatment within the first six hours after the snakebite, 13.7% within 6–12 hours and 17.9% with more than 12 hours after bite. Most of the snakebites occurred in the feet (58.8%) and legs (27.4%).

**Table 1:** Clinical and epidemiological features of 9,191 snakebite cases reported in the State of Amazonas, 2007 to 2012.

Characteristics (n; completeness)	Number	%
**Sex (n = 9,191; 100%)**		
Male	7,257	79.0
Female	1,934	21.0
**Area of occurrence (n = 9,010; 98.0%)**		
Rural	6,323	70.2
Urban	2,687	29.8
**Age group in years (n = 9,191; 100%)**		
0–15	2,216	24.1
16–45	5,019	54.6
45–65	1,603	17.4
>65	353	3.9
**Work-related accident (n = 8,200; 89.2%)**		
Yes	4,506	55.0
No	3,694	45.0
**Time-interval to medical assistance (hrs) (n = 8,612; 93.7%)**		
0–6	5,892	68.4
6–12	1,181	13.7
>12	1,539	17.9
**Anatomical site (n = 9,122; 99.2%)**		
Feet	5,372	58.8
Legs	2,498	27.4
Trunk	37	0.4
Upper limbs	1,123	12.3
Head	92	1.0
**Species (n = 8,366; 91.0%)**		
*Bothrops*	6,184	73.9
Probably *Lachesis*	1,999	23.9
*Crotalus*	37	0.5
*Micrurus*	36	0.4
Non-venomous snakebites	110	1.3
**Clinical severity of envenoming (n = 8,769; 95.4%)**		
Mild	4,040	46.1
Moderate	4,019	45.8
Severe	710	8.1
**Outcome (n = 9,182; 99.9%)**		
Discharged	9,124	93.2
Death	55	0.6
Death from other causes	3	<0.1

Snakebites were caused mainly by *Bothrops* (73.9%; 35.5 cases per 100,000 person/year) and probably by *Lachesis* (23.9%; 11.5 cases per 100,000 person/year). *Crotalus* and *Micrurus* snakes were responsible for 0.5% (0.2 cases per 100,000 person/year) and 0.4% (0.2 cases per 100,000 person/year) of the bites, respectively. Non-venomous snakebites were recorded in 1.3%.

Most cases were mild (46.1%), followed by moderate (45.8%) and severe (8.1%) cases. There were 55 deaths due to snakebites in the study period, resulting in a 0.6% lethality rate. Lethality rates were 0.7% for *Bothrops* snakebites and 0.6% for *Lachesis* snakebites. There were no records of deaths due to *Crotalus* and *Micrurus* snakebites in the study period. According to age groups, lethality rate was 0.5% for the ≤15 years group, 0.5% for the 15 to 60 years group and 2.0% for the ≥60 years group.

**Fig 2:**
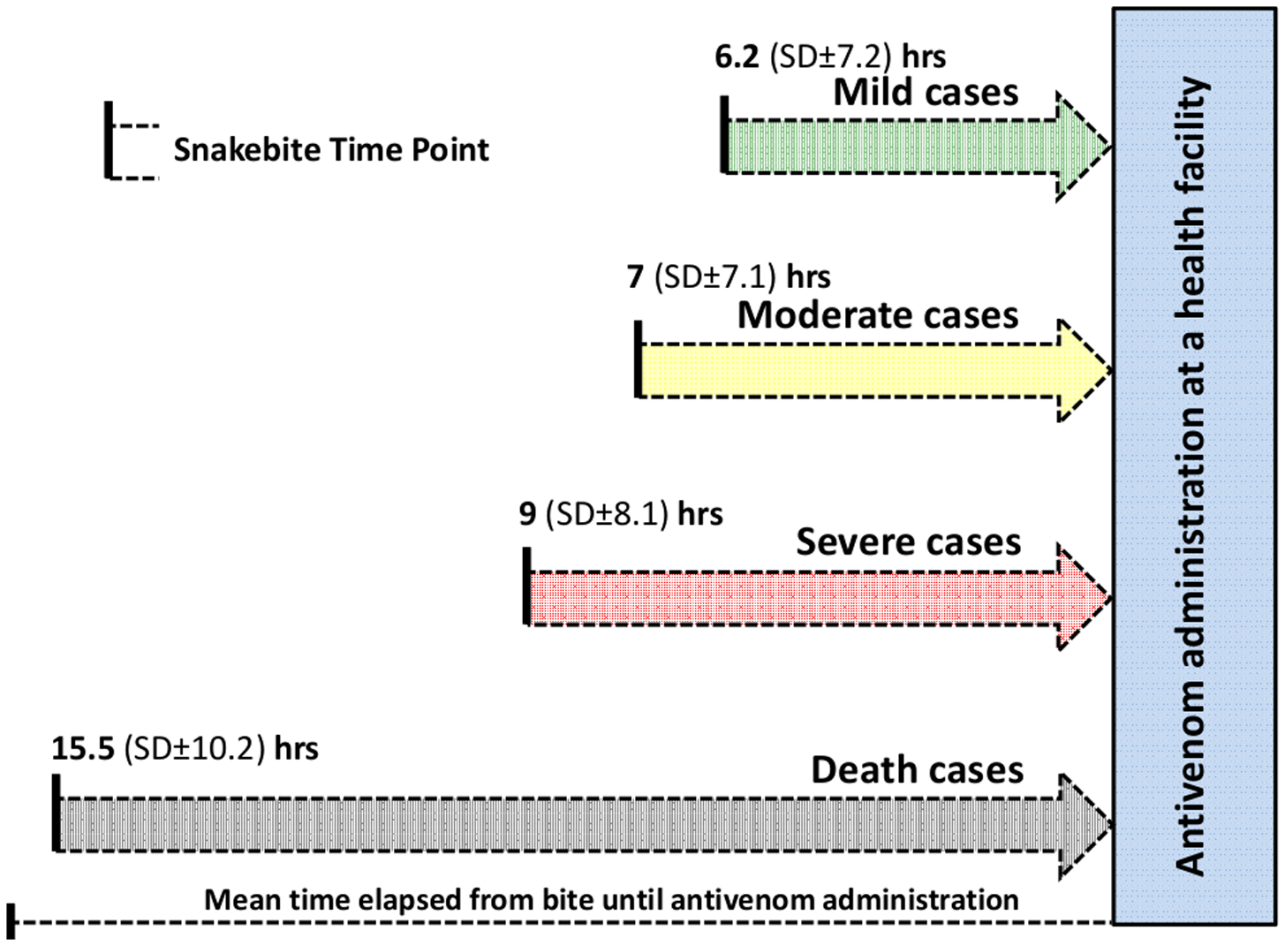
Time elapsed from bite to medical attention at a health facility according to severity grading. Average time directly associated to severity grading, with a longer time observed for cases evolving to death.

Time elapsed from bite to antivenom administration at a health facility was 6.2 (SD±7.2) hours for mild cases, 7 (SD±7.1) hours for moderate cases, 9 (SD±8.1) hours for severe cases and 15.5 hours (SD±10.2) for cases evolving to death (Fig 2).

The most frequent local manifestations were pain (97.1%), edema (85.2%), ecchymosis (15.3%) and necrosis (2.3%). Systemic manifestations were present in 19.4% of patients. Of those presenting systemic manifestations, 54.8% presented with hemorrhage, 33.7% with vagal symptoms, 20.5% with myolysis or hemolysis, 19.6% had paralysis and 12.6% had renal injury. Coagulation time was altered in 39.1% of all commers.

Antivenom was provided to 93% of *Bothrops* bites, 79% of *Crotalus* bites, 97% of *Micrurus* bites and 96% of accidents by *Lachesis*, totalling 94% of the study population. 52.7% of cases evolving to death did not receive antivenom. One per cent of accidents by *Bothrops* considered mild received less than the recommended dosage of specific antivenom, 3% of moderate cases received less than recommended and 52% of severe cases received less than expected. A total of 50% of accidents by *Lachesis* considered moderate received less than the recommended dosage and 81% of severe cases were underdosed. All accidents by *Micrurus* are reported as severe and 35% of cases received less than the recommended dose of antivenom. Lastly, for *Crotalus* injuries, 37.8% received less than the recommended dose of antivenom.

### Spatial distribution

**Fig 3:**
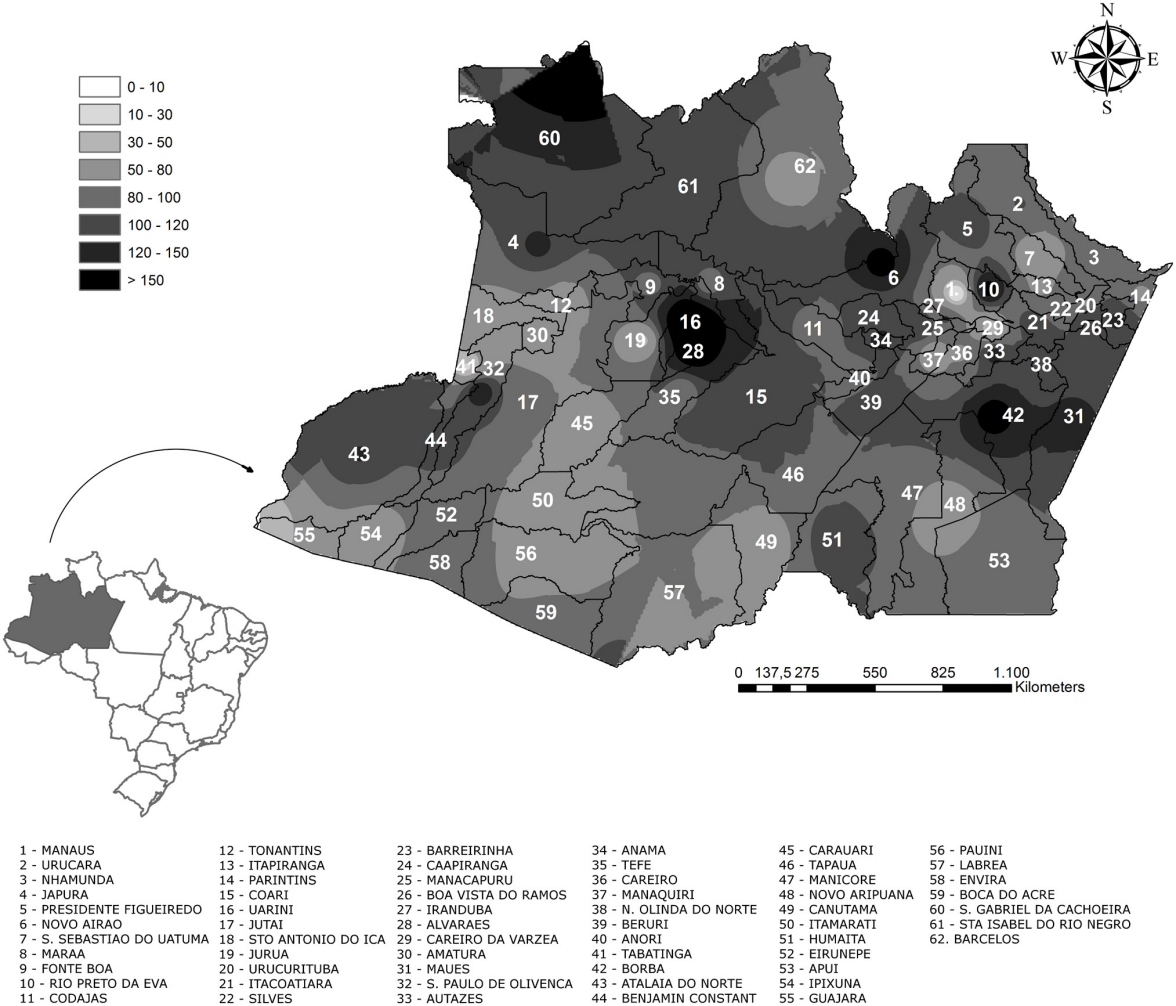
Spatial distribution of snakebites in the State of Amazonas, from 2007 to 2012. A wide area with high incidence rates extends from the Northeast to the Central region of the state, where incidence rates are ~150 cases per 100,000 inhabitants/year.

Incidence rates were unevenly distributed across the Amazonas State, although there were snakebites cases reported from all the 62 municipalities. Mapping showed a large area with high incidence rates extending from the Northeast to the Central region of the state, including three hotspots located in São Gabriel da Cachoeira (in the Colombian border), in the Uarini/Alvarães municipalities area and in Novo Airão, where incidence rates were higher than 150 cases per 100,000 inhabitants/year. Smaller areas with high snakebite incidences were located in Rio Preto da Eva, in the vicinity of the capital city, and in Borba, in the upper Southeast region of the state (Fig 3). Incidence rates by municipality are presented in S1 Table.

### Risk factors for severity and mortality

**Fig 4:**
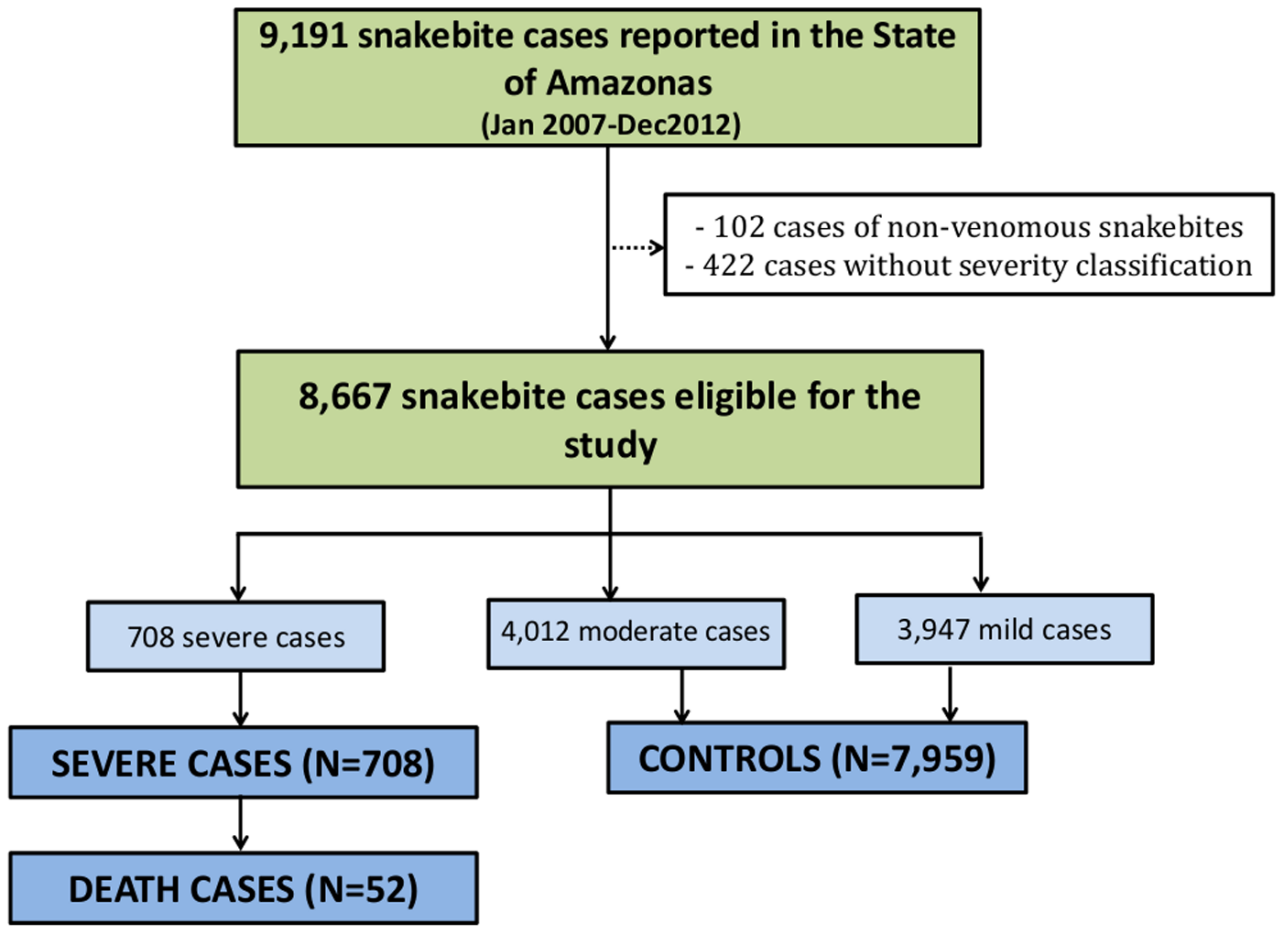
Flow chart of cases and control selection. The selection of cases and controls was based on the Brazilian Ministry of Health classification. All severe bites were included as cases, and three mild and moderate bites were in the control group for each case.

From the 9,191 snakebite cases reported in the Amazonas State in the study period, we excluded 102 cases of non-venomous snakebites and 422 cases without severity classification. We used the remaining 8,667 eligible cases to evaluate risk factors for severity and mortality. A total of 708 severe cases and 52 deaths were included as cases and 7,959 snakebites were included as controls (Fig 4).

Table 2 summarizes the results of the univariable and multivariable logistic regression models evaluating factors associated with snakebite severity. Age ≤15 years [OR = 1.26 (95% CI = 1.03–1.52); (p = 0.018)], age ≥65 years [OR = 1.53 (95% CI = 1.09–2.13); (p = 0.012)], bites related to work activities [OR = 1.39 (95% CI = 1.17–1.63); (p<0.001)], time elapsed before medical assistance >6 hours [OR = 1.73 (95% CI = 1.45–2.07); (p<0.001)] and *Micrurus* bites [OR = 5.54 (95% CI = 2.61–12.20); (p<0.001)] were independently associated with the risk of developing a severe illness after snakebites.

**Table 2:** Factors associated with snakebite severity in the State of Amazonas, 2007 to 2012.

Variables	Cases (n)	%	Controls (n)	%	Crude OR (IC 95%)	p	Adjusted OR (IC 95%)	p
**Sex**								
Male	556	78.5	6,302	79.2	0.96 (0.80–1.16)	0.680	…	…
Female	152	21.5	1,657	20.8				
**Age (years)**								
≤15	181	25.6	1,891	23.8	1.10 (0.92–1.31)	0.280	**1.26 (1.03–1.52)**	**0.018**
>15	527	74.4	6,068	76.2				
≥65	43	6.1	332	4.2	**1.48 (1.07–2.06)**	**0.017**	**1.53 (1.09–2.13)**	**0.012**
<65	665	93.9	7,659	95.8				
**Anatomical site**								
Lower limbs	558	78.8	6,357	80.0	0.93 (0.77–1.12)	0.450	…	…
Other sites	150	21.2	1,592	20.0				
**Area of occurrence**								
Rural	512	72.3	5,466	68.7	**1.19 (1.01–1.41)**	**0.045**	1.05 (0.89–1.26)	0.530
Urban	196	27.7	2,493	31.3				
**Work-related injury**								
Yes	399	56.4	3,934	49.4	**1.32 (1.13–1.54)**	**<0.001**	**1.39 (1.17–1.63)**	**<0.001**
No	309	43.6	4,025	50.6				
**Time-interval to medical assistance (hours)**								
0–6	297	41.9	2,268	28.5	**1.81 (1.55–2.12)**	**<0.001**	**1.73 (1.45–2.07)**	**<0.001**
>6	411	58.1	5,691	71.5				
**Snakebite type**								
*Bothrops*	494	75.6	5,422	74.8	1	…	…	…
Probably *Lachesis*	144	22.1	1,776	24.5	0.89 (0.73–1.08)	0.237	0.85 (0.69–1.04)	0.115
*Crotalus*	4	0.6	31	0.4	1.41 (0.49–4.02)	0.512	1.20 (0.36–3.95)	0.765
*Micrurus*	11	1.7	25	0.3	**4.82 (2.36–9.87)**	**<0.001**	**4.91 (2.30–10.47)**	**<0.001**

Table 3 compares patients evolving to death versus other patients. Age ≥65 years [OR = 3.19 (95% CI = 1.40–7.25); (p = 0.006)] and time to medical assistance >6 hours [OR = 2.01 (95% CI = 1.15–3.50); (p = 0.013)] were independently associated with risk of death.

**Table 3:** Factors associated with snakebite mortality in the State of Amazonas, 2007 to 2012.

Variables	Cases (n)	%	Controls (n)	%	Crude OR (IC 95%)	p	Adjusted OR (IC 95%)	p
**Sex**								
Male	40	76.9	6,818	79.1	0.87 (0.46–1.67)	0.695	…	…
Female	12	23.1	1,797	20.9				
**Age (years)**								
≤15	10	19.2	2,062	23.9	0.75 (0.38–1.51)	0.428	0.74 (1.35–1.56)	0.440
>15	42	80.8	6,553	76.1				
≥65	7	13.5	368	4.3	**3.48 (1.56–7.78)**	**0.001**	**3.19 (1.40–7.25)**	**0.006**
<65	45	86.5	8,247	95.7				
**Anatomical site**								
Lower limbs	43	82.7	6,882	79.9	1.20 (0.58–2.47)	0.614	…	…
Other sites	9	17.3	1,733	20.1				
**Area of occurrence**								
Rural	38	73.1	5,940	68.9	1.22 (0.66–2.26)	0.521	…	…
Urban	14	26.9	2,675	31,1				
**Work-related injury**								
Yes	24	46.2	4,309	50.0	0.85 (0.49–1.48)	0.570	…	…
No	28	53.8	4,306	50.0				
**Time-interval to medical assistance (hrs)**								
0–6	24	46.2	2,541	29.5	**2.04 (1.18–3.54)**	**0.009**	**2.01 (1.15–3.50)**	**0.013**
>6	28	53.8	6,074	70.5				
**Snakebite type**								
*Bothops*	38	77.6	5,878	75.5	1	…	…	…
Probably *Lachesis*	11	22.4	1,909	24.5	0.94 (0.48–1.83)	0.86		

## Discussion

Few previous attempts have been made to quantify the burden of envenoming resulting from snakebites in the Brazilian Amazon. Our study confirms previous hospital based reports of a wide geographical distribution of snakebites in the state of Amazonas [9]. We provide a more accurate, up-to-date estimate of the scale of the problem in the Brazilian Amazon by using official surveillance data to estimate the disease burden due to snakebites and to identify independent risk factors for severity and mortality. In this study, systemic manifestations were present in 19.4% of patients, being the most common: hemorrhage, myolysis/hemolysis, paralysis and renal injury, in agreement with previous reports from the Amazon Region, where *Bothrops atrox* is the main causal agent [18,19,30].

The mean incidence rate in the study area was 52.8 cases per 100,000 person/year, reaching more than 150 cases per 100,000 person/year in several municipalities, comparable to projections for sub-Saharan Africa and Oceania [1]. In the Brazilian Amazon, reaching health centers is difficult for riverine and indigenous populations as a result of distance and underdevelopment of means of transportation. If we account for a possible underreporting to official surveillance systems [32], the actual incidence of snakebites in this region could be among the highest in the world. We observed a large area with high incidence rates extending from the Northeast to the Central region of the state, where incidence rates were above 150 cases per 100,000 inhabitants/year. This area coincides with municipalities where Amerindian populations predominate, highlighting the vulnerability of these groups. A previous study found and association between snakebite incidence and deforestation [33]. Indeed, the hotspots identified in Novo Airão and Rio Preto da Eva, both in the Manaus region, and in Borba, correspond to areas with intense deforestation, and likely reflect the emergence of economical activities in this municipalities, namely farming and cattle raising.

Most snakebites occurred in males, between 16 and 45 years, living in rural areas and mainly related to work activities. This profile has been reported in other regions in Brazil [13,16,34,35] and in other countries [36,37] and reaffirms the strong relationship between snakebites and rural economical activities in poor regions [38], thus, negatively affecting family economies in the area. Currently, there are no interventions for primary prevention of snakebites as an occupational hazards or to speed access to health services.

More than 30% of patients took more than six hours to receive medical assistance and such delay was an independent risk factor for severity and mortality. A delay to medical care was also identified as a risk factor for poor outcomes in India [23,39], Nepal [36,40] and Nigeria [27,28]. Patients from Amazonian communities depend mainly on river transportation to reach hospitals in urban seats, causing a delay in treatment. Moreover, many victims seek medical care after a considerable delay or fail to reach a hospital in time as a result of first seeking treatment from traditional healers [9]. The lethality rate of 0.6% observed in this work is higher than some previous national reports [7,35]. We hypothesize that implementing a rapid transport system for snakebite patients would significantly reduced lethality rates as has been reported in other settings [41].

Interestingly, occupational accidents were more likely to develop severe disease. This relationship between severity and occupational exposure was also observed in India, where rural workers bitten by snakes were more prone to death [37]. We can not precisely establish a direct association between snakebite severity and occupational exposure, but the distance to be walked from the workplace in farming areas to medical care could explain this risk association. In India, patients who walked more than 1 km after the snakebite, had a delay in care and a higher risk of death [22]. Reassurance and immobilization of the affected limb with prompt transfer to a medical facility are the cornerstones of the immediate care of snakebites [29,42].

In this work, age less than 15 years old was also an independent risk factor for severity, probably as a result of smaller body volumes [2]. This risk factor was also observed in India [22] and Turkey [43]. One half of deaths from snakebites in California affected children under 5 years of age [44].

Age ≥65 years was a risk factor for severity and mortality. This is relevant as literature scarcely reports elderly as being at a higher risk. In *Bothrops* and *Crotalus* snakebites reported in São Paulo, Brazil, patients ≥50 years-old had a higher mortality when compared to younger patients [45]. Taking into account that most snakebite complications, especially by *Bothrops*, are due to acute renal failure [46–50], the greater severity associated with the elderly may be related to higher prevalences of chronic comorbidities (i.e. hypertension and diabetes) that can lead to a predisposition to evolve to necrosis and acute kidney injury after a bite [51,52]. One especulates that comorbidities leading to vascular disease, such as hypertension and diabetes, commonly found in the elderly, may precipitate life-threatening complications after snakebite envenomings. In a case series from Brazil, eight of 309 patients (2.6%) developed a cerebrovascular event after *Bothrops* envenoming; five of the eight patients died despite therapy, and the remaining three had irreversible sequelae [53]. Cases of stroke related to *Bothops* envenomings have been reported in Brazil in the elderly, including in the Amazon region [54,55]. Population aging will likely affect snakebite epidemiology and its public health impact thus requiring preparation by local health services with a wider distribution of antivenom and life-sustaining supportive therapies besides health care workers’ training in the management of envenoming in this neglected age group.

Our study reports significant underdosing of antivenom in the Brazilian Amazon as has been previously observed [32]. This has several possible explanations: we used an epidemiologic surveillance system to obtain our data where clinical and serum administration dosing information is collected hours or even days after the event, possibly reflecting a difficulty predicting the amount of antivenom needed upon admission; another possible explanation is the lack of appropriate training in antivenom dosing in medical personnel in remote settings; or possibly, unavailability of serum in rural areas were most of accidents occur. Underdosing may play a role in morbidity and mortality associated with snakebites in the region, exemplified by the fact that more than half of deaths lacked antivenom administration; this represents a missed opportunity to improve snakebite outcomes in the region.

Record keeping may have been influenced by the nature of the surveillance system. Some patients with mild bites in inaccessible areas may not be reported to hospitals and those evolving to severity may die on the way before reaching medical attention. In Brazil, antivenoms are prescribed exclusively by physicians in hospital settings. However, only a few patients bring the snakes for identification to the health unit, therefore the clinical-epidemiological classification is subject to misclassification, as is mostly based on clinical grounds. For instance, in the absence of the *Lachesis* or *Bothrops-Lachesis* antivenoms or in case of snake misclassification, health services often use *Bothrops* antivenom in the treatment of laquetic accident. However, previous studies in the same region have shown that *Bothrops* antivenom was ineffective to neutralize the coagulant activity of *L*. *muta* venom in humans, hence, contributing to severity [56]. In an uncertain proportion, completion of severity grading may occur retrospectively, according to the number of antivenom vials administered. Since patients are commonly seen initially at the emergency room, case notification mostly occurs after antivenom therapy and sometimes even after patient discharge. As a result, severity classification of snake envenoming by healthcare providers depends on the quality of this retrospective information. As all *Micrurus* envenomings are considered potentially severe by the Brazilian Ministry of Health guidelines, it was expected for this type of envenoming to be associated with severity. In fact, it was an expected result resulting from an biased association, rather than a real risk factor for poor outcome.

In conclusion: i) the incidence of snakebites in the Western Brazilian Amazon is among the highest in the world; ii) snakebites show a wide distribution in this region; iii) snakebites represent an occupational health problem for rural populations and public heath measures are needed to prevent occupational snakebites; iv) age ≤15 years and ≥65 years, work related bites and delayed medical treatment >6 hours were independently associated with the risk of developing severity and mortality; age ≥65 years and time to medical assistance >6 hours were independently associated with mortality. These features highlight the need for public health interventions aiming to decrease time to medical attention in vulnerable groups. In the Amazon, a major concern relates to the failure in antivenom distribution and storage. The lack of an adequate cold chain impairs antivenom distribution to rural areas and may result in loss of material. Moreover, there is a lack of systematic professional training on diagnosis, specific therapy and clinical management of complications [32]. Improvements in antivenom distribution and time to medical attention could have a significant impact in patient outcomes.

## Supporting Information

S1 File Clinical grading of snakebite according to the Brazilian Ministry of Health. (DOCX)

S2 File STROBE Statement: Checklist of items that should be included in reports of case-control studies. (DOC)

S1 Table Annual mean incidence of snakebites by municipality in the State of Amazonas, 2007–2012. (DOC)
