# Supplementary material for: Older Age and Time to Medical Assistance Are Associated with Severity and Mortality of Snakebites in the Brazilian Amazon: A Case-Control Study
Source: PLoS One. 2015 Jul 13;10(7):e0132237. doi: 10.1371/journal.pone.0132237 (PMC4500501; doi:10.1371/journal.pone.0132237)
Supplement: S1 File — (DOCX) [file pone.0132237.s001.docx]

**Supplementary Box 1.** Clinical grading of snakebite according to the Brazilian Ministry of Health [1,2].

| **Type of snakebite** | **Antivenom** | **Clinical grading** | **Number of antivenom ampoules to be administered** |
| --- | --- | --- | --- |
| *Bothrops* | SAB^b^, SABL^c^ or SABC^d^ | **Mild**: pain, local swelling and bruising | 2-4 |
|  |  | **Moderate**: pain, evident swelling and bruising, minor systemic bleeding phenomena | 4-8 |
|  |  | **Severe**: intense local changes, severe bleeding, hypotension/shock, acute renal failure, anuria | 12 |
| *Lachesis*^a^ | SABL | **Moderate**: pain, swelling, blistering; there may be bleeding without vagal manifestations | 10 |
|  |  | **Severe**: pain, swelling, blistering, bleeding, abdominal cramps, diarrhea, bradycardia, hypotension | 20 |
| *Crotalus* | SAC^e^ or SABC | **Mild**: ptosis, slight blurred vision of late onset, without changing the color of urine, mild or absent myalgia | 5 |
|  |  | **Moderate**: ptosis, slight blurred vision of early onset, discrete myalgia, dark urine | 10 |
|  |  | **Severe**: ptosis, obvious and intense blurred vision, intense and generalized myalgia, dark urine, oliguria or anuria | 20 |
| *Micrurus* | SAEla^f^ | It is recommended to consider all cases as potentially severe due to the risk of respiratory failure | 10 |

^a^ Due to the potential severity of *Lachesis* bites, they are considered clinically moderate or severe, and there are no mild cases. Diagnosis is mainly clinical and epidemiological, with no routine laboratory test to confirm the type of circulating venom. For areas where there is overlap in the geographical distribution of snakes *Bothrops* and *Lachesis*, such as in the Brazilian Amazon, the differential diagnosis is possible only with the identification of the animal or, in the case of *Lachesis* bites, the possibility of development of vagal manifestations [1,2]; ^b^ SAB=anti-*Bothrops* serum; ^c^ SABL=anti-*Bothrops* plus anti-*Lachesis* serum; ^d^ SABC=anti-*Bothrops* plus anti-*Crotalus* serum; ^e^ SAC= anti-*Crotalus* serum; ^f^ SAEla= anti-elapidic serum (for *Micrurus* bites).

1. Brazilian Ministry of Health (2001). Manual de diagnóstico e tratamento de acidentes por animais peçonhentos. Brasília: Brazilian Ministry of Health. 120 p.
2. Brazilian Ministry of Health (2009) Caderno 14-Acidentes por Animais Peçonhentos. Guia de vigilância epidemiológica. Brasília: Brazilian Ministry of Health. p 23.
