# Supplementary material for: Older Age and Time to Medical Assistance Are Associated with Severity and Mortality of Snakebites in the Brazilian Amazon: A Case-Control Study
Source: PLoS One. 2015 Jul 13;10(7):e0132237. doi: 10.1371/journal.pone.0132237 (PMC4500501; doi:10.1371/journal.pone.0132237)
Supplement: S1 Table — (DOC) [file pone.0132237.s003.doc]

**Supplementary Information 2**

Supplementary Table. Annual mean incidence of snakebites by municipality in the State of Amazonas, 2007-2012.

| **Municipality** | **Cases** | **Cases/year** | **Population estimated in 2010** | **Incidence per year/100,000 inhabitants** |
| --- | --- | --- | --- | --- |
| **Alvarães** | 131 | 26.2 | 14,088 | 186.0 |
| **Amaturá** | 33 | 6.6 | 9,467 | 69.7 |
| **Anamã** | 67 | 13.4 | 10,214 | 131.2 |
| **Anori** | 68 | 13.6 | 16,317 | 83.4 |
| **Apuí** | 83 | 16.6 | 18,007 | 92.2 |
| **Atalaia do Norte** | 92 | 18.4 | 15,153 | 121.4 |
| **Autazes** | 210 | 42.0 | 32,135 | 130.7 |
| **Barcelos** | 83 | 16.6 | 25,718 | 64.6 |
| **Barreirinha** | 172 | 34.4 | 27,355 | 125.8 |
| **Benjamin Constant** | 194 | 38.8 | 33,411 | 116.1 |
| **Beruri** | 93 | 18.6 | 15,486 | 120.1 |
| **Boa Vista do Ramos** | 90 | 18.0 | 14,979 | 120.2 |
| **Boca do Acre** | 135 | 27.0 | 30,632 | 88.1 |
| **Borba** | 304 | 60.8 | 34,961 | 173.9 |
| **Caapiranga** | 56 | 11.2 | 10,975 | 102.1 |
| **Canutama** | 36 | 7.2 | 12,738 | 56.5 |
| **Carauari** | 78 | 15.6 | 25,744 | 60.6 |
| **Careiro** | 125 | 25.0 | 32,734 | 76.4 |
| **Careiro da Várzea** | 41 | 8.2 | 23,930 | 34.3 |
| **Coari** | 382 | 76.4 | 75,965 | 100.6 |
| **Codajás** | 97 | 19.4 | 23,206 | 83.6 |
| **Eirunepé** | 125 | 25.0 | 30,665 | 81.5 |
| **Envira** | 80 | 16.0 | 16,338 | 97.9 |
| **Fonte Boa** | 109 | 21.8 | 22,817 | 95.5 |
| **Guajará** | 21 | 4.2 | 13,974 | 30.1 |
| **Humaitá** | 260 | 52.0 | 44,227 | 117.6 |
| **Ipixuna** | 72 | 14.4 | 22,254 | 64.7 |
| **Iranduba** | 208 | 41.6 | 40,781 | 102.0 |
| **Itacoatiara** | 507 | 101.4 | 86,839 | 116.8 |
| **Itamarati** | 25 | 5.0 | 8,038 | 62.2 |
| **Itapiranga** | 29 | 5.8 | 8,211 | 70.6 |
| **Japurá** | 46 | 9.2 | 7,326 | 125.6 |
| **Juruá** | 24 | 4.8 | 10,802 | 44.4 |
| **Jutaí** | 78 | 15.6 | 17,992 | 86.7 |
| **Lábrea** | 203 | 40.6 | 37,701 | 107.7 |
| **Manacapuru** | 504 | 100.8 | 85,141 | 118.4 |
| **Manaquiri** | 67 | 13.4 | 22,801 | 58.8 |
| **Manaus** | 698 | 139.6 | 1,802,014 | 7.8 |
| **Manicoré** | 208 | 41.6 | 47,017 | 88.5 |
| **Maraã** | 78 | 15.6 | 17,528 | 89.0 |
| **Maués** | 334 | 66.8 | 52,236 | 127.9 |
| **Nhamundá** | 80 | 16.0 | 18,278 | 87.5 |
| **Nova Olinda do Norte** | 158 | 31.6 | 30,696 | 102.9 |
| **Novo Airão** | 123 | 24.6 | 14,723 | 167.1 |
| **Novo Aripuanã** | 54 | 10.8 | 21,451 | 50.4 |
| **Parintins** | 389 | 77.8 | 102,033 | 76.3 |
| **Pauini** | 54 | 10.8 | 18,166 | 59.5 |
| **Presidente Figueiredo** | 145 | 29.0 | 27,175 | 106.7 |
| **Rio Preto da Eva** | 218 | 43.6 | 25,719 | 169.5 |
| **Santa Isabel do Rio Negro** | 102 | 20.4 | 18,146 | 112.4 |
| **Santo Antônio do Içá** | 86 | 17.2 | 24,481 | 70.3 |
| **São Gabriel da Cachoeira** | 444 | 88.8 | 37,896 | 234.3 |
| **São Paulo de Olivença** | 221 | 44.2 | 31,422 | 140.7 |
| **São Sebastião do Uatumã** | 28 | 5.6 | 10,705 | 52.3 |
| **Silves** | 38 | 7.6 | 8,444 | 90.0 |
| **Tabatinga** | 72 | 14.4 | 52,272 | 27.6 |
| **Tapauá** | 93 | 18.6 | 19,077 | 97.5 |
| **Tefé** | 269 | 53.8 | 61,453 | 87.6 |
| **Tonantins** | 56 | 11.2 | 17,079 | 65.6 |
| **Uarini** | 143 | 28.6 | 11,891 | 240.5 |
| **Urucará** | 86 | 17.2 | 17,094 | 100.6 |
| **Urucurituba** | 69 | 13.8 | 17,837 | 77.4 |
| **Total** | **9,174** | **1,835.2** | **3,483,955** | **52.68** |
| Cases occurred outside the Amazonas State but notified in this state | 17 |  |  |  |
